# Prophylactic Injection of Recombinant Alpha-Enolase Reduces Arthritis Severity in the Collagen-Induced Arthritis Mice Model

**DOI:** 10.1371/journal.pone.0136359

**Published:** 2015-08-24

**Authors:** Clément Guillou, Céline Derambure, Manuel Fréret, Mathieu Verdet, Gilles Avenel, Marie-Laure Golinski, Jean-Christophe Sabourin, François Le Loarer, Sahil Adriouch, Olivier Boyer, Thierry Lequerré, Olivier Vittecoq

**Affiliations:** 1 INSERM, U905, Rouen, France; 2 Normandy University, Institute for Research and Innovation in Biomedicine (IRIB), Rouen, France; 3 Rouen University Hospital, Department of Rheumatology, Rouen, France; 4 Rouen University Hospital, Department of Pathology, Rouen, France; 5 Rouen University Hospital, Department of Immunology, Rouen, France; Wayne State University, UNITED STATES

## Abstract

**Objective:**

To evaluate the ability of the glycolytic enzyme alpha-enolase (ENO1) or its immunodominant peptide (pEP1) to reduce the severity of CIA in DBA/1 mice when injected in a prophylactic way.

**Methods:**

Mice were treated with mouse ENO1 or pEP1 one day prior to collagen II immunization. Clinical assessment was evaluated using 4 parameters (global and articular scores, ankle thickness and weight). Titers of serum anti-ENO1, anti-cyclic citrullinated peptides (anti-CCP) and anti-CII (total IgG and IgG1/IgG2a isotypes) antibodies were measured by ELISA at different time-points. Disease activity was assessed by histological analysis of both anterior and hind paws at the end of experimentation.

**Results:**

Prophylactic injection of 100 μg of ENO1 reduced severity of CIA. Serum levels of anti-CII antibodies were reduced in ENO1-treated mice. Concordantly, ENO1-treated mice joints presented less severe histological signs of arthritis. ENO1 did not induce a shift toward a Th2 response since IgG1/IgG2a ratio of anti-CII antibodies remained unchanged and IL-4 serum levels were similar to those measured in the control group.

**Conclusions:**

Pre-immunization with ENO1 or its immunodominant peptide pEP1 reduces CIA severity at the clinical, immunological and histological levels. Effects of pEP1 were less pronounced. This immunomodulatory effect is associated with a reduction in anti-CII antibodies production but is not due to a Th1/Th2 shift.

## Introduction

Rheumatoid arthritis (RA) is an autoimmune and inflammatory disorder characterized by chronic inflammation and synovial hyperplasia, leading to destruction of the cartilage and bone. RA is a chronic disease whose etiology is still unclear and largely multifactorial. Indeed, RA pathogenesis involves an interaction between environmental (notably Tobacco exposure and *Porphyromonas gingivalis* infection) [1], genetic (HLA-DRB1*0401 and *0404, PTPN22) [2], hormonal [3] and immunological factors [4]. Among them, the autoimmune response plays a pivotal role, notably through the production of autoantibodies directed against citrullinated proteins (ACPAs) [5].These autoantibodies can be detected several years before the onset of clinical features. As shown by van de Stadt *et al*. [6], ACPA epitope spreading occurs over a period of several years prior to the onset of clinical RA. Studies of the fine specificity of this family of autoantibodies revealed a vast array of antigenic determinants in several proteins such as fibrinogen, vimentin, collagen and alpha-enolase (ENO1) [7, 8].

Autoantibodies targeting ENO1 are found in sera from patients with very early RA [9]. Complementary studies have shown that citrullination of ENO1 is crucial for its autoantigenicity since 46% of tested RA sera showed reactivity against the citrullinated form while only 13% recognized the non-citrullinated form [10]. To date, the presence of autoantibodies against citrullinated ENO1 remains exclusive of RA. Several arguments suggest that they have a pathogenic role in this disease. A mechanism of molecular mimicry has been highlighted. An ENO1 immunodominant peptide was identified as Citrullinated Enolase Peptide 1 (CEP-1). Antibodies to this epitope are observed in 37–62% of sera from RA patients. This peptide has 82% homology with that from *Porphyromonas gingivalis*, and the levels of antibodies against CEP-1 correlate with those of antibodies against the bacterial peptide [11]. Further, antibodies to the human peptide cross-react with citrullinated recombinant *Porphyromonas gingivalis* enolase [12]. This suggests a role for bacterial infection in priming autoimmunity in a subset of RA patients.

ENO1 is a multifaceted protein expressed in various tissues such as liver, kidney but also synovial tissue. This protein can be found in different forms and in different cell compartments. The cytoplasmic form represents a highly conserved glycolytic enzyme. Cell surface ENO1 has been discovered to act as a plasminogen receptor, suggesting a role in modulating the fibrinolytic system in RA pathophysiology [13]. Furthermore, nuclear Myc binding protein 1 (MBP-1), an alternative translation transcript of *Eno1* gene, functions as a transcriptional repressor of the c-myc proto-oncogene, and thereby regulates cell growth and differentiation [14].

To determine the pathophysiological mechanisms involved in RA, several murine models have been developed. Collagen-induced arthritis (CIA) is the most widely used model since it shares many clinical, immunological and histopathological features with the human disease [15]. Several studies have already used the CIA model in order to elucidate the role of different auto-antigens in the RA pathogenesis. Some of them have evaluated the effects of the citrullinated form of autoantigens when injected in a prophylactic way whereas others have focused on their native form. In this regard, citrullinated proteins have discordant effects in the CIA model. Indeed, mice pre-treated with a citrullinated peptide derived from fibrinogen before induction of CIA demonstrated significantly reduced arthritis severity and incidence compared with controls [16]. In contrast, pre-treament with a citrullinated form of BiP, a chaperonin protein known as a putative antigen in RA, exacerbated CIA [17]. However, *in vivo* study of BiP pre-immunized CIA mice showed immunomodulatory properties [18], such as a decrease of anti-CII antibodies titers, a shift toward a Th2 cytokine profile (IL-4) and the induction of regulatory T cells [19]. In the DR4-IE-transgenic mice model, arthritis is induced by immunization by either the citrullinated or the native form of human ENO1 as well as of *P*. *gingivalis* ENO1 [20].

To our knowledge, no data are available to date concerning the prophylactic effects of the native form of ENO1 or pEP1 in the CIA model. The purpose of the present study was to evaluate the potential immunomodulatory effects of ENO1 and of its immunodominant peptide in the CIA model.

## Materials and Methods

### Recombinant mouse ENO1 and synthetic non-citrullinated EP1 production

*Eno1* cDNA was obtained by RT-PCR from RNA extracted from mouse liver. Then, it was cloned in a plasmidic expression vector (pET15b) harboring a histidine tag. After DNA sequence verification, the plasmid was transferred into *Escherichia coli*. Protein production and purification were performed by Proteogenix (Oberhausbergen). This recombinant ENO1 (GI: 158853992) was diluted in urea buffer (2 M urea, 50 mM Phosphate, 5 mM HCl-Tris) for solubilization. We then checked that the protein has not been carbamylated using a commercial kit (OxiSelect Protein Carbamylation ELISA Kit, Cell Biolabs). Finally, as expected, the protein was not functional since it was unable to catalyse the transformation of 2-phospho-pyruvate into phosphoenolpyruvate as in the glycolytic pathway.

Since lipopolysaccharides (LPS), components of GRAM negative bacteria such as *E*. *coli*, may interfere with our experiments, we removed LPS from ENO1 extract by affinity chromatography (Endotrap, Profos AG). LPS titration was performed using a commercial kit (Qcl chromogenic LAL kit, Lonza). This procedure allowed elimination of 99.5% of LPS without loss of ENO1. The final titer of LPS was below the level of detection (<1 EU/mL).

The immunodominant non-citrullinated peptide from *P*. *gingivalis* (pEP1) whose sequence is “CKIIGREILDSRGNPTVEC” was produced by the same manufacturer (Proteogenix, Oberhausbergen).

### Collagen-Induced Arthritis

Collagen-induced arthritis was performed as referred in Kim et al. [21]. At day 0, DBA/1 male mice (age 6 weeks), obtained from Janvier (Le Genest Saint Isle) were injected subcutaneously at the tail base with 100 μg of bovine CII (Chondrex) emulsified in complete Freund’s adjuvant (CFA). A booster injection of 100 μg of CII in incomplete Freund’s adjuvant (IFA) was performed subcutaneously at day 21. All animal experiments were conducted in accordance with institutional guidelines. The agreement numbers of the animal facilities are B76-450-05, B76-540-7 and experimentator is A.76-91. This study has been approved by the ethics committee (Comité Regional d’éthique en Expérimentation Animale Normandie) with the following numbers N/02-12-10/24/12-13.

### Pre-treatment of CIA mice with recombinant ENO1 or pEP1

One day before immunization with type II collagen, different doses of ENO1 (10 or 100 μg) were injected intraperitoneally in mice in a total volume of 100 μl per mice. In addition, Bovine Serum Albumin (BSA) (100 μg) was administered as a control. We also tested different doses of pEP1 (10 or 100 μg) in the same conditions that ENO1. Control peptide was a random sequence of 19 amino acids. Each group comprised 10 mice.

### Mice follow-up and arthritis evaluation

Evaluation of arthritis was based on clinical, immunological and histological parameters over 80 days. Clinical evaluation was monitored twice a week. Blood samples were drawn twice a month. Histological analysis of joints was performed at the end of experimentation.

### Clinical assessment

Clinical evaluation was performed in double-blind conditions and was determined by 4 scores: global score (0 = normal paw; 1 = moderate redness and swelling from tarsal (or carpal) joint or digits; 2 = moderate redness and swelling from tarsal (or carpal) joint to half paw; 3 = severe redness and swelling from tarsal (or carpal) to metatarsal joints), articular score on each articulation (0 = normal joint (carpal, tarsal and digits); 1 = redness; 2 = swelling; 3 = deformation; 4 = necrosis), ankle thickness with an appropriate caliper and weight follow-up.

### Immunological evaluation

Sera were collected from mice and stored at -80°C until analysis.

Several markers were measured at different time points (day 0, 15, 30, 45, 60) during follow-up: anti-CCP, anti-ENO1 or anti-pEP1, and anti-CII IgG antibodies.

Anti-ENO1 or anti-pEP1 IgG antibodies titers were measured by homemade ELISA. 96-well plates were coated with 1 μg/well of ENO1. After saturation (PBS1X, Tween 0.05%, SVF 5%), 50 μL of mouse serum were incubated for 1h. Then, we used mouse anti-IgG HRP-conjugated antibodies (LifeTechnologies) for 1 h. TMB (Sigma) was used to reveal the presence of anti-ENO1. To prepare the standard, BALB/C mice have been immunized with ENO1 or pEP1 and polyclonal anti-ENO1 or anti-pEP1 antibodies have been purified from ENO and pEP1 immunized mice.

Anti-CII IgG antibodies levels were measured by Mouse IgG anti-Bovine Collagen Type II ELISA (Morwell Diagnostics GMBH) according to the manufacturer’s instructions. Two different isotypes (IgG1 and IgG2a) of anti-CII antibodies have also been assessed by home-made ELISA using anti-IgG1-HRP (dilution 1:5000) or anti-IgG2a-HRP (dilution 1:5000) conjugated antibodies (LifeTechnologies). Anti-CII IgG1 (Abcam) and IgG2a (Thermofisher) antibodies were used as standard. Serum dilution was optimized.

Serum levels of a panel of cytokines were measured by Luminex Performance Assay, Mouse cytokine premixed kit (R&D Systems) according to the manufacturer instructions. This Luminex Assay measures the levels of the following cytokines: IFNα, IL-1β, IL-2, IL-4, IL-6, IL-10, IL-17 and TNFα.

### Histological analysis

Mice were sacrificed by cervical dislocation at the end of experimentation. For each animal, both hind and anterior paws were dissected and fixed for 48 hours in 4% phosphate-buffered formaldehyde. After fixation, samples were decalcified in DC3 decalcifier (Labonord) for 36 hours and embedded in paraffin blocks. Eigth μm-thick tissue sections were stained with haematoxylin, eosin and safranin. Histologic examination was carried out blindly on 2 semi-serial sections of both anterior and hind paws in the pathology department from the Rouen University Hospital.

Cell infiltration in synovial tissue as well as cartilage and juxta-articular bone destruction were graded from 0 to 3 according to the procedure detailed by Larsson et al. [22]. Briefly, on each histological slice, 4 scores were measured: inflammatory score (0 = no inflammatory cells in the joint cavity; 1 = a few inflammatory cells in the joint cavity; 2 = joint cavity partly filled with inflammatory cells; 3 = joint cavity totally filled with inflammatory cells), synovitis score (0 = healthy, uninflamed appearance of the synovium; 1 = mild thickening of the synovium; 2 = substantial thickening of the synovium; 3 = severe thickening of the synovium), cartilage destruction score (0 = normal appearance; 1 = minor destruction of the cartilage surface; 2 = clear loss of cartilage; 3 = cartilage almost absent in the whole joint), bone destruction score (0 = normal appearance; 1 = minor signs of destruction; 2 = up to 30% destruction; 3 = more than 30% destruction). Histological analysis was performed by a trained pathologist unaware of the received treatment.

### Statistical analysis

Two-ways ANOVA followed by Bonferonni post-test was used for comparison of clinical or biological data between ENO1 (or pEP1) and control groups. T-test was used for histological analysis.

Correlations between histological findings (synovitis, *pannus* formation, cartilage and bone erosion), biological and clinical data was studied using Spearman’s rank correlation coefficient.

## Results

### Prophylactic injection of ENO1 or pEP1 reduces severity of arthritis in the CIA mouse model

**Fig 1:**
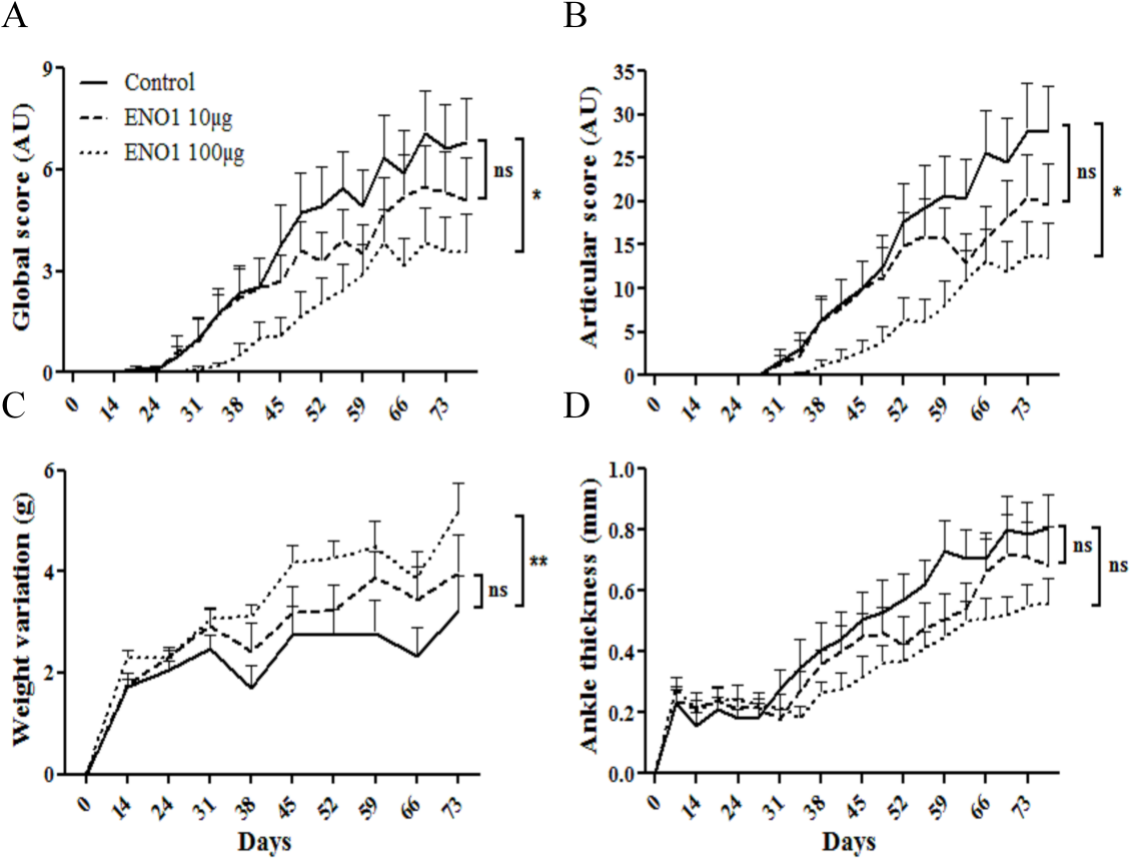
Prophylactic injection of recombinant ENO1 reduces arthritis severity in the collagen-induced arthritis mouse model. 100**μ**g CII was injected to DBA/1 mice at day 0 in CFA, a boost was performed 21 days later in IFA. Recombinant enolase (ENO1) or control (BSA) was injected subcutaneously one day before the first immunization with CII. For ENO1, two doses were tested (10**μ**g = ENO1 10**μ**g (n = 10) and 100**μ**g = ENO1 100**μ**g (n = 10)). For control mice (n = 10), BSA (100 **μ**g) was injected in the same buffer as the one used for ENO1 buffer. Clinical status was evaluated by global score (A), articular score (B), weight variation (C) and ankle thickness (D). Bars show the mean ± SEM; * = p < 0.05; ** = p < 0.01 by 2way ANOVA and Bonferroni post-test.

To investigate the effects of ENO1, DBA/1 mice were injected intraperitoneally with 10 or 100 μg ENO1 or BSA (as control) 24 hours before immunization with type II collagen. While the lowest dose (10 μg) of ENO1 did not modulate the severity of arthritis, the highest dose of ENO1 (100 μg) significantly reduced the severity of arthritis both for global score (p<0.05) (Fig 1A), articular score (p<0.05), (Fig 1B) and weight variation (p<0.001) (Fig 1C) while there was only a trend in the reduction of ankle thickness (p = 0.09) (Fig 1D).

**Fig 2:**
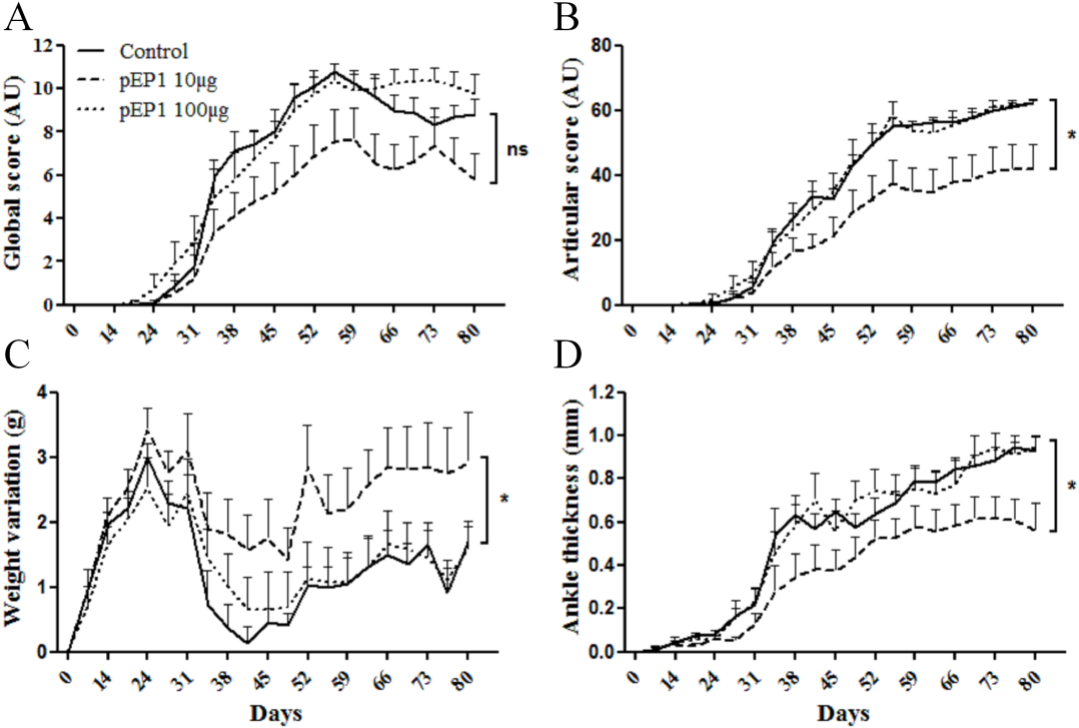
Prophylactic injection of the immunodominant epitope of ENO1 from *P*. *gingivalis* (pEP1) reduces arthritis severity in the collagen-induced arthritis mouse model. 100**μ**g CII was injected to DBA/1 mice at day 0 in CFA, a boost was performed 21 days later in iFA. pEP1 or its control (peptide of 19 aminoacid with random sequence) were injected intraperitoneally one day before the first immunization with CII. Two doses were tested (10**μ**g = pEP1 10**μ**g (n = 10) and 100**μ**g = pEP1 100**μ**g (n = 10)). Clinical status was evaluated by global score (A), articular score (B), weight variation (C) and ankle thickness (D). Bars show mean ± SEM; * = p < 0.05, by 2way ANOVA and Bonferroni post-test.

Since pEP1 is the immunodominant epitope of ENO1, we also assessed its influence on arthritis severity when injected prophylactically. A dose of 10 μg of pEP1 was able to significantly reduce arthritis severity according to the different parameters analyzed: global score (p<0.05) (Fig 2A), articular score (p<0.05) (Fig 2B), tarsal thickness (p<0.05) (Fig 2C) and weight variation (p<0.05) (Fig 2D). In contrast, the highest (100 μg) dose of pEP1 had no significant effect as illustrated in Fig 2.

### Prophylactic injection of ENO1 reduces the production of anti-collagen II antibodies

**Fig 3:**
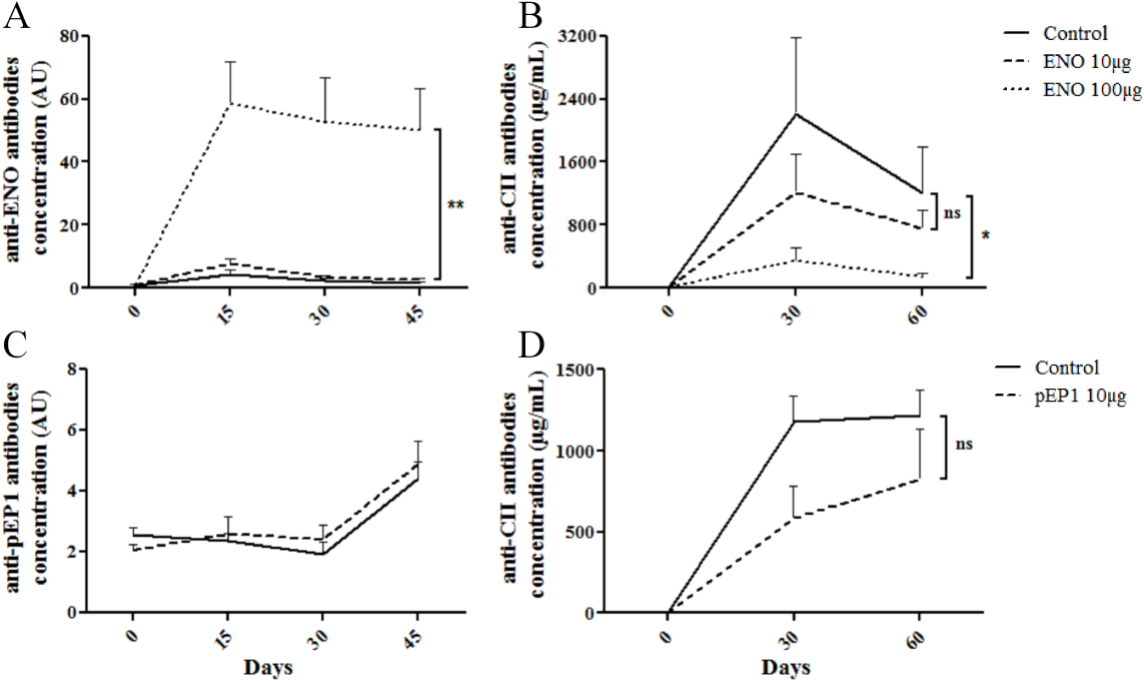
Antibody production after prophylactic injection of ENO1 or pEP1 in CIA mice model. Serum levels of anti-ENO1 (A), anti-pEP1 (C) and anti-collagen II (B and D) antibodies in control or treated mice were measured by ELISA. AU is considered as Arbitrary Units. Bars represent the mean ± SEM; * = p < 0.05; ** = p < 0.01, by 2way ANOVA and Bonferroni post-test (A and B); * = p < 0.05, by Mann-Withney (C and D).

To evaluate the impact of prophylactic injection of ENO1 or pEP1 on the autoimmune response, we measured the levels of different autoantibody populations referred to as anti-ENO1 or anti-pEP1, anti-CII and anti-CCP antibodies. Prophylactic injection of 100 μg ENO1 induced a production of anti-ENO1 antibodies (p<0.001) (Fig 3A). No antibodies production was observed when using 10μg of the protein. In a dose dependent manner, the titers of anti-CII antibodies were significantly lower with the highest dose of ENO1 (p<0.05) (Fig 3B). For immunological analysis of pEP1, we studied only the dose with a clinical effect *i*.*e*. 10 μg. Regardless of injected doses, prophylactic injection of pEP1 did not elicit the production of anti-pEP1 antibodies (Fig 3C). Conversely, the impact of pEP1 on the production of autoantibody was less pronounced. Indeed, injection of 10 μg of pEP1 reduces the production of anti-CII antibodies but not significantly (p = 0.1) (Fig 3D).

### IgG1/IgG2a ratio of anti-CII antibodies is unchanged in ENO1 and pEP1 treated mice

**Fig 4:**
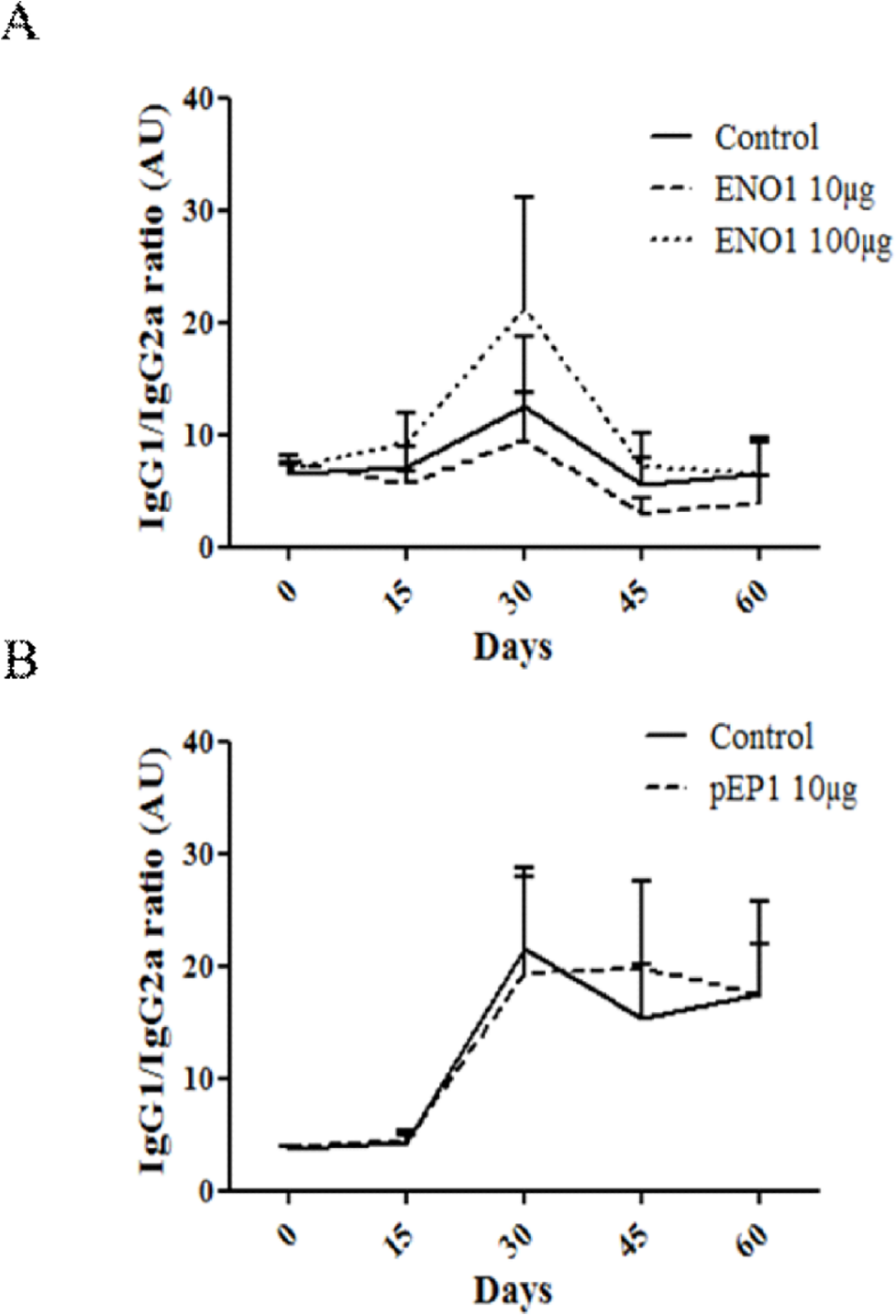
Antibody production after prophylactic injection of ENO1 or pEP1 in CIA mice model. Serum levels of anti-CII IgG1 and IgG2a isotypes were measured by ELISA and IgG1/IgG2a ratio was calculated (A and B). Serum was obtained at different time points after the first CII immunization. Bars represent the mean ± SEM. Statistical analysis used are 2way ANOVA (A) or Mann-Withney (B).

In order to investigate whether ENO1 could favor a Th2 or Th1 cellular response, we measured the levels of anti-CII IgG1 and IgG2a antibodies. Although the production of anti-CII antibodies was reduced in mice treated with ENO1 or pEP1, the IgG1/IgG2a ratio remained unmodified in both experiments (Fig 4A and 4B). Levels of anti-CCP antibodies were also measured but no production was observed in our different experiment settings (data not shown).

### Unchanged serum levels of IL-4 and IL-6 in ENO1 or pEP1 treated mice

**Fig 5:**
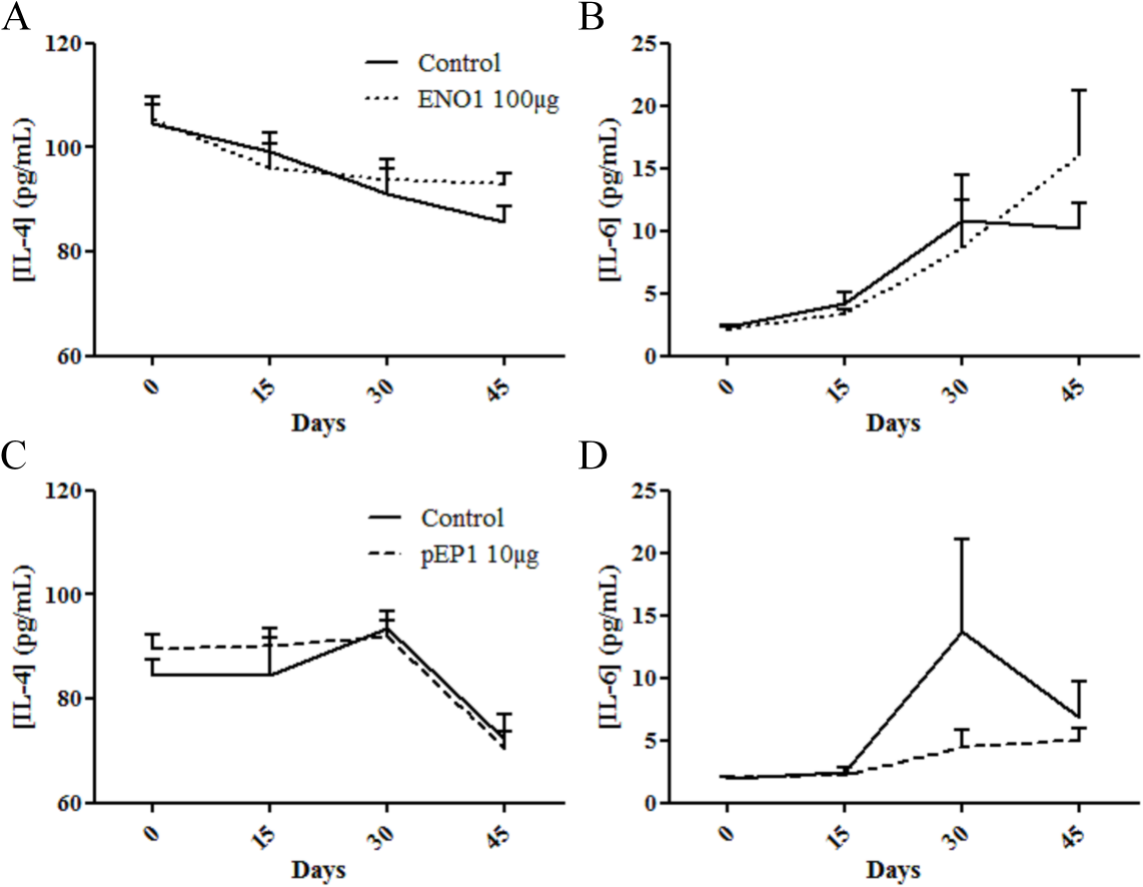
No difference in serum levels of IL-4 and IL-6 in ENO1 or pEP1 treated mice compared to control mice. Serum levels of IL-4 (A and C) and IL-6 (B and D) in ENO1 treated mice (A-B) and pEP1 treated mice (C-D), compared to control mice, were measured by Luminex. Bars represent the mean ± SEM. Statistical analysis used are 2way ANOVA (A and B) or Mann-Withney (C and D).

Since prophylactic injections of ENO1 or pEP1 led to prevention of arthritis severity in the CIA model, one of the hypotheses was that this molecule has the ability to induce immunomodulatory cell populations. To test this hypothesis, we evaluated at different time-points a panel of pro- and anti-inflammatory cytokines in mice sera using a multiplex assay. Among the 8 cytokines tested, only IL-4 and IL-6 were detectable in mice sera. However, the serum levels of IL-4 and IL-6 in ENO1 or pEP1 treated mice were comparable to those observed in the control mice (Fig 5).

### Prophylactic injection of ENO1 reduces histological scores

**Fig 6:**
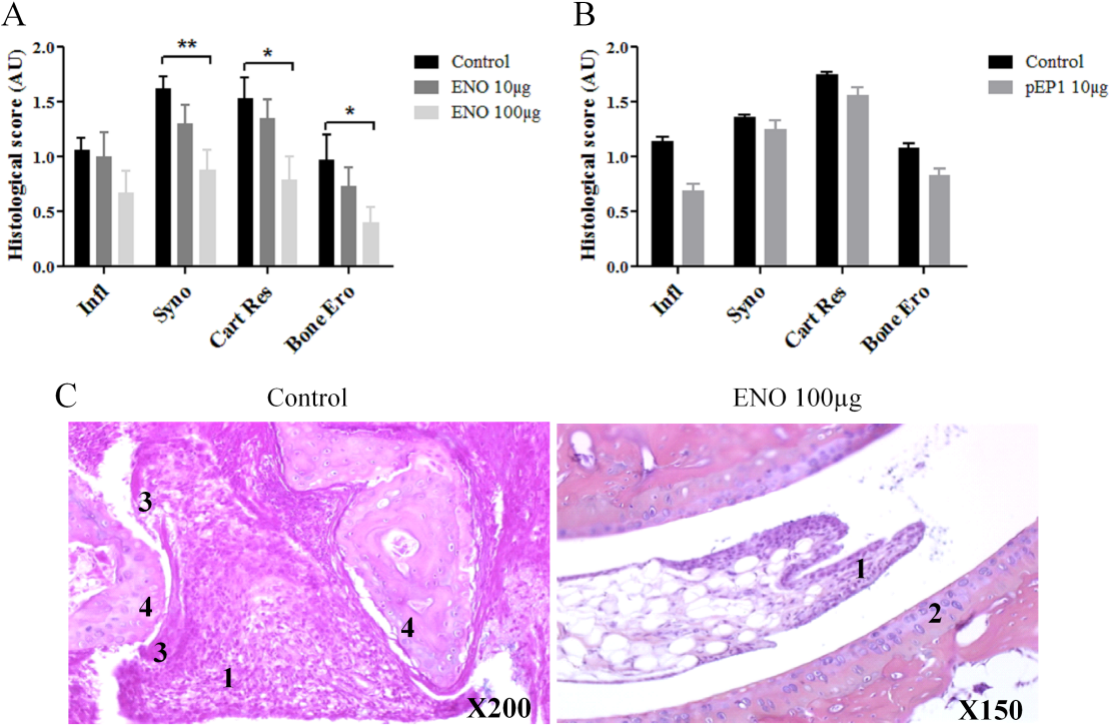
Prophylactic injection of ENO1 but not pEP1 reduces arthritis and joint destruction in the CIA mouse model. Histological analyses were performed on CIA mice receiving prophylactic injection of ENO1 (A) or pEP1 (B) and were compared to those carried out in controls. Both hind and anterior paws were dissected and fixed for 48h in 10% phosphate-buffered formaldehyde 80 days after CII immunization. Different scores were assessed: inflammation (Infl), synovitis (Syno), cartilage resorption (Cart Res) and bone erosion (Bone Ero). In C, anterior paws of control mice (left) and anterior paws of CIA mice who received ENO1 100**μ**g in a prophylactic way (right) were represented. 1: Synovial tissue hypertrophy; 2: Newly formed cartilage; 3: Fibrin deposit; 4: Cartilage resorption. Histograms show mean ± SEM; * = p < 0.05; ** = p < 0.01 by t-tests.

Histological analysis was performed on hind or foreleg of each mouse 80 days after CII immunization. Four scores were assessed: inflammatory score, synovitis score, cartilage destruction score, bone destruction score. First, compared to control, 100 μg ENO1 was able to reduce pannus formation and joint damage as demonstrated by lower scores of synovitis (p<0.01), cartilage resorption (p<0.05) and bone erosion (p<0.05). However, no significant effect was observed in terms of inflammation score (p = 0.15) (Fig 6A). These findings were not found with the dose of 10 μg ENO1 (Fig 6A). Concerning the impact of pEP1 on histological parameters, there was no significant impact on each score (Fig 6B). In fact, as usual in CIA model, we observed, in the control group, a strongly active arthritis with inflammatory infiltrates, and bone and cartilage resorption due to synovial hypertrophy (Fig 6C left). In the group receiving 100 μg ENO1, although synovial tissue hypertrophy was observed in most joints, there was no sign of activity and new cartilage formation was observed (Fig 6C right).

### Correlation between clinical assessment and histological analysis

As shown in Table 1, a significant correlation was found between clinical and histological scores for experiments based on prophylactic injection of ENO1 compared to prophylactic injection of control.

**Table 1:** Correlation between clinical and histological scores for experiments based on prophylactic injection of ENO1. Correlations between histological findings and clinical data were studied using Spearman’s rank correlation coefficient. Data are represented as Rs and p value.

		Inflammation	Synovitis	Cartilage Resorption	Bone Erosion
Global Score	R square	0.52	0.32	0.52	0.47
	P value	<0.0001	0.0006	<0.0001	<0.0001
Articular Score	R square	0.32	0.34	0.6	0.56
	P value	0.0005	0.0004	<0.0001	<0.0001
Ankle Thickness	R square	0.2	0.23	0.41	0.36
	P value	0.0088	0.0045	<0.00001	0.0002
Weight Variation	R square	0.26	0.35	0.53	0.49
	P value	0.0025	0.0003	<0.0001	<0.0001

## Discussion

This study shows for the first time that, when injected in a prophylactic way in the CIA model, ENO1 has a dose-dependent inhibitory effect on arthritis severity, as assessed by different clinical, immunological and histological parameters. The effects of pEP1 were less pronounced. The preventive effect of ENO1 was associated to a reduction in the production of anti-CII antibodies. The protocol used with a single injection of ENO1 or pEP1 24 hours prior to immunization with CII was the most effective tested protocol. Indeed, various schemes of administration have been tested including intravenous or subcutaneous injections of ENO1 or pEP1, with or without IFA, 7 or 14 days before the first injection of CII. None of these protocols showed comparable efficacy to the protocol performed in this study (data not shown).

We and others have previously shown that the autoantibody repertoire in early untreated RA is directed against several categories of antigens, notably some enzymes of the glycolytic pathway such as aldolase, PGK1, glucose-6-phosphate isomerase and ENO1, and chaperones molecules including calreticulin, HSP-60 and BiP [23]. For some of them (fibrinogen, vimentin…), citrullination is involved in the breakdown of immunological tolerance observed in RA patients. However, the pattern of reactivity is variable among the panel of autoantigens. Indeed, they can be recognized either in their citrullinated or their native form, while some of them, such as BiP and ENO1, are the target of autoantibodies that recognize both their citrullinated and native forms. In this regard, autoantibodies to native BiP and ENO1 are readily detected in RA patients at a frequency of 63% and 25% respectively [9, 24]. As suggested by some reports [17], the two autoantibody populations, *i*.*e*. directed against the citrullinated and native forms of these two proteins, are certainly the consequence of different processes in RA patients. Notably, the production of antibodies recognizing the native form may be the consequence of an overexpression of the antigen, as observed for BiP in the inflamed synovial tissue [24].

Both forms of autoantigens are likely to be implicated in the pathophysiology of RA. Nevertheless, there are discordances in arthritis development in mouse models after immunization with citrullinated antigens. In the context of a specific HLA background, as observed in the DR4-IE transgenic mouse model for instance, the citrullinated forms of fibrinogen and ENO1 were demonstrated to be arthritogenic [25]. In contrast, other authors claimed that human and mouse citrullinated ENO1 are immunogenic while they may fail to induce arthritis even in genetic backgrounds susceptible to CIA [26]. Yet, the pathogenicity of immune reactivity against some antigens is not restricted to their citrullinated form. For instance, in the K/BxN mouse model, arthritis is induced by pathogenic autoantibodies directed against glucose-6-phosphate isomerase [27]. Moreover, immunization with G6PI induces a severe polyarthritis in normal mice [28].

In the present study, we show that another enzyme of the glycolytic pathway, ENO1, has the ability to reduce arthritis severity in the CIA model when injected in a prophylactic way 24 hours before immunization with type II collagen. In this experiment, particular attention was given to the antigen preparation and particularly in the absence of residual contaminants such as LPS and of posttranslational modification favored by urea containing buffer that might be responsible for carbamylation of the protein. In the present study, urea containing buffer or LPS alone did not provide any beneficial effect on arthritis severity (data not shown). Thus, the immunomodulatory dose-dependent effects observed herein, both at clinical (Figs 1 and 2) and histological levels (Fig 6) can be reliably ascribed to mouse ENO1. Our data are consistent with those observed by the group of Panayi which has shown that another RA autoantigen, BiP, also had immunomodulatory properties when injected prophylactically in its recombinant unmodified form in the CIA mouse model [19].

To a lesser degree, pEP1 was also able to reduce arthritis severity when injected in the same conditions than ENO1 (Figs 1 and 2). However, effects were less pronounced, notably on histological scores (Fig 6). One explanation may be that we have evaluated the effects of the immunodominant epitope recognized by autoantibodies directed against citrullinated ENO1. This peptide is possibly less relevant for inducing an immune response leading to reduction of arthritis severity. Besides, as mentioned previously, we have shown that the reactivity against peptides derived from ENO1 was directed against some peptides that are not related to citrullination [23]. Furthermore, EP1 has shown a specific association with HLA-DR4 alleles. Herein, pEP1 has not been evaluated in DBA/1 mice with a specific type II major histocompatibility complex background.

Unlike pEP1, ENO1 was immunogenic in this model since all mice showed a strong IgG response to mouse ENO1 (Fig 3A). Pre-immunization with ENO1 had no impact on ACPA production since their titers were comparable between treated and control groups. ACPA titers are rather low during the course of CIA in DBA/1 mice. In the same way, low ACPA response was observed in DBA/1 mice immunized with fibrinogen [29]. Interestingly, ENO1 injected prophylactically was able to reduce the production of anti-CII IgG autoantibodies that are well-known to be pathogenic in the CIA model (Fig 3B). Indeed, anti-CII IgG antibodies can directly induce arthritis when transferred to DBA/1 mice [30]. Besides, anti-citrullinated fibrinogen antibodies can enhance tissue injury in CIA when coadministered with a dose of anti-CII antibodies, suggesting a pivotal role of anti-CII autoantibodies in the pathophysiology of CIA [16].

Ability of antigen-specific immune tolerization to induce immune deviation has been illustrated both in animal models and in humans. In the CIA model, prophylactic effects of recombinant native BiP are associated with the emergence of regulatory T-cells secreting IL-4 and IL-10, suggesting an immune deviation towards a Th2 response [19]. Moreover, dnaJP1, a dominant epitope from another heat shock protein, dnaJ, can induce an immune deviation in effector T cells in RA patients through a qualitative change from a pro-inflammatory phenotype to a more tolerogenic phenotype characterized by a decline in the production of TNF-α and a corresponding increase of IL-10 [31]. In the present study, a mechanistic hypothesis for ENO1 tolerization was that ENO1 may induce a shift from Th1 to Th2 responses. However, study of the anti-CII IgG1/IgG2a ratio was not in favor of a Th2 immune deviation in the process described herein (Fig 4) and the serum level of IL-4 was unchanged in treated mice compared to control over the follow-up period (Fig 5). In the present study, two main mechanisms might be advanced to explain the effects observed in the treated mice. The first one would be the emergence of a regulatory cell population such as Treg or Breg cells while the second one might be the consequence of the production of “protective” anti-ENO1 antibodies. In pEP1 treated mice, the effects are less pronounced at the clinical and biological levels since the decreased rate of anti-CII antibodies is not statistically significant. We can thus postulate that both mechanisms mentioned previously could be involved in mice treated with the whole molecule whereas only the first one would be concerned in mice treated with the peptide. That would explain the difference observed in terms of degree of response between ENO1 and pEP1. The regulatory cell population remains to be determined. In this respect, it was shown that BIP, another major RA autoantigen, when injected according to the same procedure than that reported herein, was able to prevent arthritis severity in CIA via the induction of regulatory T cells secreting IL-4 [19]. Furthermore, it would be interesting to study more specifically the anti-ENO1 antibodies and their potentially protective role because some studies on the prevention and treatment of autoimmune diseases have shown the effectiveness of protocols using different types of antibodies, either bispecific or multimers, including some that have been assessed in the CIA mouse model [32–35]. Other mechanisms are not excluded since the beneficial effects of the P140 phosphopeptide, issued from the spliceosomal U1-70 K small nuclear ribonucleoprotein, in MRL/lpr mice, might be explained by impairment of the autophagic pathway and endogenous MHC II presentation [36]. Taken together those data, we can speculate that ENO1 probably displays a higher number of effects on the immune system than pEP1, leading to specifically lower levels of anti-CII antibodies. Other investigations are needed to determine the underlying mechanisms leading to a protective effect of ENO1 in the CIA model. Antigen- or peptide-driven therapies appear to be promising approaches. Data from collagen II or P140 based approaches in arthritis- or lupus-prone mice are convincing [37]. The mode of antigen or peptide administration is certainly of great importance since intravenous or subcutaneous injection of ENO1 had no effect in contrast to intraperitoneal route in the present study. This is in accordance with findings obtained with P140 since only its IV injection (but not intranasal or subcutaneous administration) resulted in a significant delay of signs classically associated to lupus in pre-autoimmune MRL/lpr mice [38]. Intraperitoneal or IV routes appear more effective than a subcutaneous injection in inducing “tolerance” [39]. Other strategies also exist like the induction of a specific tolerance such as eye-induced tolerance or oral tolerance [40–43]. It seems interesting to explore the full potential of ENO1 by the induction of such tolerance against ENO1 in CIA model using different methods, and to compare and contrast their effects with those obtained by using our method described in this study. Together, the data reported herein demonstrate for the first time an immunomodulatory effect provided by ENO1 when injected in a prophylactic way in the CIA model. A similar strategy has recently been proposed in recent-onset type 1 diabetes using glutamic acid decarboxylase [44]. Also, the Lupuzor/P140 peptide was able to significantly improve clinical and biological status of patients with systemic lupus erythematosus in a randomized, double blind, placebo-controlled phase IIb clinical trial [45]. Hence, we believe that the present results may pave the way towards the potential development of an antigen- or peptide-driven therapy for patients with RA.
